# Health Related Quality of Life in Adult Low and High-Grade Glioma Patients Using the National Institutes of Health Patient Reported Outcomes Measurement Information System (PROMIS) and Neuro-QOL Assessments

**DOI:** 10.3389/fneur.2019.00212

**Published:** 2019-03-15

**Authors:** Nicolette Gabel, David B. Altshuler, Amanda Brezzell, Emily M. Briceño, Nicholas R. Boileau, Zachary Miklja, Karen Kluin, Thomas Ferguson, Kaitlin McMurray, Lin Wang, Sean R. Smith, Noelle E. Carlozzi, Shawn L. Hervey-Jumper

**Affiliations:** ^1^Department of Physical Medicine and Rehabilitation, University of Michigan, Ann Arbor, MI, United States; ^2^Department of Neurosurgery, University of Michigan, Ann Arbor, MI, United States; ^3^Department of Neurology, University of Michigan, Ann Arbor, MI, United States; ^4^Department of Speech-Language Pathology, University of Michigan, Ann Arbor, MI, United States

**Keywords:** glioma, health-related quality of life, language, neuro-rehabilitation, PROMIS, Neuro-QOL, astrocytoma, glioblastoma

## Abstract

Health related quality of life (HRQOL) measures have become increasingly important in the management of glioma patients in both research and clinical practice settings. Functional impairment is common in low-grade and high-grade glioma patients as the disease has both oncological and neurological manifestations. Natural disease history as well as medical or surgical treatment can negatively influence HRQOL. There are no universal standards for HRQOL assessment in glioma patients. In this study, we examine patient perspectives on functional outcome domains and report the prevalence of impairments rates using the National Institutes of Health (NIH) Patient Reported Outcomes Measurement Information System (PROMIS) and Neuro-QOL item banks as measures of HRQOL. Retrospective analysis of a prospectively collected dataset involving 79 glioma patients reveals that quality of life concerns are the most important consideration behind making decisions about treatment in 80.7% of patients. The prevalence of functional impairment by PROMIS and NEURO-QOL assessment is high, ranging from 28.6% in the physical function domain to 43.9% in the cognitive function domain. Pain and anxiety related to physical decline is higher in LGG patients compared to HGG patients. Aphasia severity also impacts HRQOL. The results of this study suggest that the PROMIS and NEURO-QOL assessments may be important HRQOL metrics for future use in larger clinical research and clinical trial settings.

## Introduction

Gliomas are the most frequent primary brain tumor in adults (1). There are currently more than 700,000 people living with a primary central nervous system tumor in the United States. Despite relatively low incidence, gliomas result in a disproportionate share of cancer morbidity and mortality. Brain tumors account for the highest number of years of life lost when compared to non-CNS cancers (2). Despite treatment with maximal safe surgical resection with or without adjuvant chemoradiation, overall survival has remained largely unchanged. Survival is approximately 14 months for glioblastoma and 6 to 15 years for those with WHO II and III glioma (LGG) depending on the genetic profile of the tumor.

Health-related quality of life (HRQOL) metrics have become increasingly important in brain tumor research alongside standard patient outcome measures such as progression-free and overall survival. There are several validated HRQOL assessments used in clinical practice and clinical trials research. Continued efforts to develop and implement HRQOL measurements are needed as research study and clinical endpoints. The relationship between HRQOL and survival in adult glioma is poorly understood. The World Health Organizations' (WHO) International Classification of Functioning Disability and Health (WHO 2010) defines HRQOL based on the following functional domains: physical, social, emotional well-being, and relational (3–5). In the glioma patient population, both disease progression and treatment related effects have been shown to negatively impact HRQOL (6–9).

While HRQOL metrics continue to become incorporated in clinical practice and clinical trials research, there is no consensus regarding assessment measures. The objective of this study was to evaluate patient perspectives on functional domain affecting health related quality of life. We then applied the National Institutes of Health (NIH) Patient Reported Outcomes Measurement Information System (PROMIS) and Quality of Life in Neurological Disorders (Neuro-QOL) instruments as subjective HRQOL patient-reported outcome (PRO) measures in an adult low- and high-grade glioma patient population. Although there are some overlapping domains, PROMIS was developed for use across the general population with multiple chronic health conditions, whereas Neuro-QoL was focused on developing measures that represent HRQOL domains that are specific to neurological disorders (specifically stroke, Parkinson's disease, multiple sclerosis, child and adult epilepsy, amyotrophic lateral sclerosis, and muscular dystrophy). Therefore, the calibration samples and content, while often overlapping, are different for each tool. These tools, PROMIS and Neuro-QoL, may be useful in assessing patient HRQOL and may be an important component of a multidisciplinary treatment approach for glioma patients. Many HRQOL factors may be common to patients with any cancer diagnosis (e.g., pain, emotional distress, sleep disturbance, etc.) and adequately assessed with PROMIS measures; yet, some domains of HRQOL are likely to be uniquely impacted by neurological changes associated with glioma, including cognitive and behavioral functioning in daily life, and may therefore be better captured by Neuro-QOL. To the authors' knowledge this is the first study employing the use of PROMIS and Neuro-QOL prospectively in a cohort of adult low and high-grade gliomas.

## Methods

The study design involved retrospective analysis of a prospectively collected HRQOL single institution data registry. Participants were recruited at the time of an initial clinic visit following the diagnosis of a presumed glioma. Patients remained enrolled in the study after histopathologic confirmation of a new WHO grade I–IV glioma. Exclusion criteria included age <18 and language and/or neurocognitive dysfunction limiting patient ability to complete PROMIS and Neuro-QOL questionnaires. Aphasia was assessed by the Boston Diagnostic Aphasia Examination (BDAE).

All patients were administered the Montreal Cognitive Assessment (MoCA), a screening instrument developed to estimate global cognitive ability in the service of detecting mild cognitive impairment and dementia (10). Several studies have demonstrated its utility in brain tumor populations, wherein it has been shown to have superior sensitivity compared to other screening instruments (11), is correlated with quality of life measures (12), and predicts median overall survival (13).

To examine patient preferences on functional domains and HRQOL, structured interviews were conducted focusing on how patients frame functional and cognitive domains with their disease experiences based on methodology established by Mortensen and Jakobsen (14, 15). Analysis of these semi-structured interviews was used to identify those functional domains considered important to individual glioma patients, which were then developed into a study questionnaire using a Likert scale to identify each domain as extremely important, important, neutral, somewhat important, or not at all important.

Study participants completed PROMIS version 1.0 and Neuro-QOL version 1.1 as HRQOL measures. Examined HRQOL functional domains included Neuro-QOL cognition, PROMIS physical functioning, and PROMIS ability to participate in social roles and activities; impairment domains include PROMIS pain, sleep, fatigue, depression, anxiety, and Neuro-QOL emotional/behavioral dyscontrol. PROMIS Physical Function assesses self-reported (not actual) ability to perform with one's lower extremities (e.g., walking), upper extremities (e.g., dexterity), back, and neck, and to engage in instrumental activities of daily living (16). PROMIS Anxiety assesses anxiety symptoms, including hyperarousal and fear (17, 18). PROMIS Depression measures feelings of worthlessness and sadness among other symptoms of depression. PROMIS Fatigue measures the intensity and impact of fatigue on quality of life (17, 18). PROMIS Sleep disturbances measures perceived quality, adequacy, and satisfaction with sleep as well as difficulties falling asleep and staying asleep (19, 20). PROMIS Ability to participate in social roles and activities measures one's reported ability to participate and be involved in social roles and activities (17, 18). PROMIS Pain interferences assesses the impact of pain on physical, emotional, and recreational activities (17, 18). PROMIS Pain intensity instrument assesses how much a patient hurts. The pain intensity short form is global (i.e. not site specific) and universal rather than disease specific (16). Neuro-QoL Cognitive function measures perceived executive functioning and memory difficulties (20, 21). Neuro-QOL Emotional and behavioral dyscontrol assesses emotionality and impulsivity (20, 21) Normalized mean t-scores for each domain were standardized to 50. For functional domains, higher scores indicate less distress (score >50 more desirable); for impairment domains, higher scores indicate more distress, higher scores indicate more distress (score <50 more desirable). Analyses were conducted using SAS 9.4 statistical software. Independent *t*-tests were conducted to assess differences between LGG (WHO grades I-II) and HGG (WHO grades III-IV) groups. Prevalence of impairment was assessed in the study population where patients who scored >1 standard deviation beyond the normative mean was considered impaired.

Language assessments were performed by a certified Speech pathologist using the Boston Diagnostic Aphasia Examination Severity rating (BDAE) (22, 23). All assessments were performed in a noise controlled clinical examination room according to standard protocol. BDAE aphasia severity scores reflect the ability to communicate wants, needs, ideas with or without help from listener. BDAE severity scores ranging from 1 to 2 were considered severe aphasia (1 = severe, 2 = moderately-severe). Scores 3–5 were categorized as mild-moderate aphasia (3 = moderate, 4 = mild, 5 = trace) (22). Study inclusion required BDAE aphasia severity score ≥1 (22, 23).

Univariate analyses were conducted to assess differences between HGG and LGG for each of the PROMIS and Neuro-QOL measures. Partial eta-squared (η^2^) effect sizes were examined to determine the proportion of variance in HRQOL that was accounted for by tumor-grade (small = 0.01, moderate = 0.09, and large = 0.25) (24). An independent t-test was performed to compare PROMIS and Neuro-QOL scores among patients according to language dysfunction categorized as mild aphasia (BDAE 3-5) vs. severe aphasia (BDAE 1–2).

## Results

Seventy-nine patients were eligible for inclusion. Of the 79 patients, 58 had HGG and 21 had LGG. Average patient age was 52 years (SD = 15.6). Global cognitive status was not different between LGG and HGG patient cohorts by the Montreal Cognitive Assessment (MOCA) (mean HGG = 21.8, LGG = 24.8; *P* = 0.114). Additional population characteristics are found in Table 1.

**Table 1:** Patient demographics and clinical characteristics.

**Variable**	**High-grade glioma (*n* = 58)**	**Low-grade glioma (*n* = 21)**	**All (*N* = 79)**	***p*-value**
Mean age, years (SD)	55.2 (15.0)	42.7 (13.6)	51.9 (15.6)	**0.01**
Mean body mass index (SD)	28.5 (5.8)	30.3 (5)	29.0 (5.6)	0.25
**Gender (%)**				0.06
Female	24 (41.4)	5 (23.8)	29 (36.7)	
Male	34 (58.6)	16 (76.2)	50 (63.3)	
**Education (%)**				0.56
Completed college	48 (82.8)	15 (71.4)	63 (79.7)	
Did not complete college	10 (17.2)	6 (28.6)	16 (20.3)	
Employment at time of diagnosis (%)				0.31
Employed	27 (46.6)	12 (57.1)	40 (50.6)	
Unemployed	31 (53.4)	9 (42.9)	39 (49.4)	
**Handedness (%)**				0.35
Right-handed	51 (87.9)	21 (100.0)	72 (91.1)	
Left-handed	6 (10.3)	0 (0.0)	6 (7.6)	
Both	1 (1.7)	0 (0.0)	1 (1.3)	
**Smoking status (%)**				0.34
Smoker	4 (6.9)	3 (14.3)	7 (8.9)	
Non-smoker	54 (93.1)	18 (85.7)	72 (91.1)	
**Major presenting symptom (%)**				0.60
Cognitive dysfunction	14 (24.1)	4 (19.0)	18 (22.8)	
Headaches	4 (6.9)	2 (9.5)	6 (7.6)	
Incidental	3 (5.2)	4 (19.0)	7 (8.9)	
Aphasia	10 (17.2)	2 (9.5)	12 (15.2)	
Weakness	8 (13.8)	2 (9.5)	10 (12.7)	
Seizure	19 (32.8)	7 (33.3)	26 (32.9)	
**Tumor location (%)**				0.22
Frontal	19 (32.8)	5 (23.8)	28 (35.4)	
Parietal	14 (24.1)	2 (9.5)	16 (20.3)	
Temporal	13 (22.4)	4 (19.0)	17 (21.5)	
Occipital	2 (3.4)	1 (4.8)	3 (3.8)	
Insular	6 (10.3)	4 (19.0)	10 (12.7)	
Other (thalamus, brainstem, cerebellum)	4 (6.9)	5 (23.8)	9 (11.4)	
**Tumor side (%)**				0.37
Left	29 (50.0)	9 (42.9)	38 (48.1)	
Midline	5 (8.6)	2 (9.5)	7 (8.9)	
Right	24 (41.4)	10 (47.6)	34 (43.0)	

*Bold values mean significant p value (<0.05)*.

### Patient Perspectives on Functional Domains of Importance

Study subjects were surveyed about the importance of quality of life concerns vs. survival on medical decision making at the time of diagnosis. Among LGG patients, 81.3% indicated treatment strategies based on quality of life concerns, while 78.6% of HGG patients were concerned primarily with quality of life (*P* = 0.69) (Table 2). The functional domains of greatest concern and importance were language, motor, and memory. There were no differences between LGG and HGG patients in these domains; however, HGG patients placed higher importance on creativity/problem-solving and art domains compared to LGG patients (*P* = 0.009).

**Table 2:** Functional domain of importance and quality of life concerns influencing medical decision-making in adult patients with low- or high-grade glioma.

	**High-grade glioma**						**Low-grade glioma**						**All**						
	***n***	**Mean**	**SD**	**SE**	**95% CI Lower**	**95% CI Upper**	***n***	**Mean**	**SD**	**SE**	**95% CI Lower**	**95% CI Upper**	***n***	**Mean**	**SD**	**SE**	**95% CI Lower**	**95% CI Upper**	***p*-value**
**FUNCTIONAL DOMAIN**																			
Mathematical abilities	58	**3.2**	1.35	0.21	2.78	3.62	21	2.75	1.34	0.34	2.04	3.47	79	**3.08**	1.35	0.18	2.72	3.43	0.258
Language	58	**4.43**	0.86	0.13	4.16	4.70	21	4.56	0.73	0.18	4.18	4.95	79	**4.47**	0.82	0.11	4.25	4.68	0.583
Motor abilities	58	**4.38**	1.06	0.16	4.05	4.71	21	4.25	1.00	0.25	3.72	4.78	79	**4.35**	1.04	0.14	4.07	4.62	0.671
Attention and distractibility	58	**4.12**	0.97	0.15	3.82	4.42	21	4.06	0.85	0.21	3.61	4.52	79	**4.10**	0.93	0.12	3.86	4.35	0.838
Memory	58	**4.24**	1.03	0.16	3.92	4.56	21	4.06	0.85	0.21	3.61	4.52	79	**4.19**	0.95	0.13	3.93	4.45	0.547
Coordination of movement	58	**4.14**	0.90	0.14	3.86	4.42	21	4.00	1.03	0.26	3.45	4.55	79	**4.10**	0.93	0.12	3.86	4.35	0.606
Music	58	**3.29**	1.27	0.20	2.89	3.68	21	2.75	1.34	0.34	2.04	3.47	79	**3.14**	1.30	0.17	2.80	3.48	0.164
Art	58	**2.91**	1.34	0.21	2.49	3.32	21	1.94	0.77	0.19	1.53	2.35	79	**2.64**	1.28	0.17	2.30	2.98	**0.009**
Creativity and problem-solving	58	**4.10**	0.88	0.14	3.82	4.37	21	3.31	1.45	0.36	2.54	4.08	79	**3.88**	1.11	0.15	3.59	4.17	**0.015**

*Bold values mean significant p value (<0.05)*.

### HRQOL Functional and Impairment Domains Using PROMIS and Neuro-QOL

**Figure 1:**
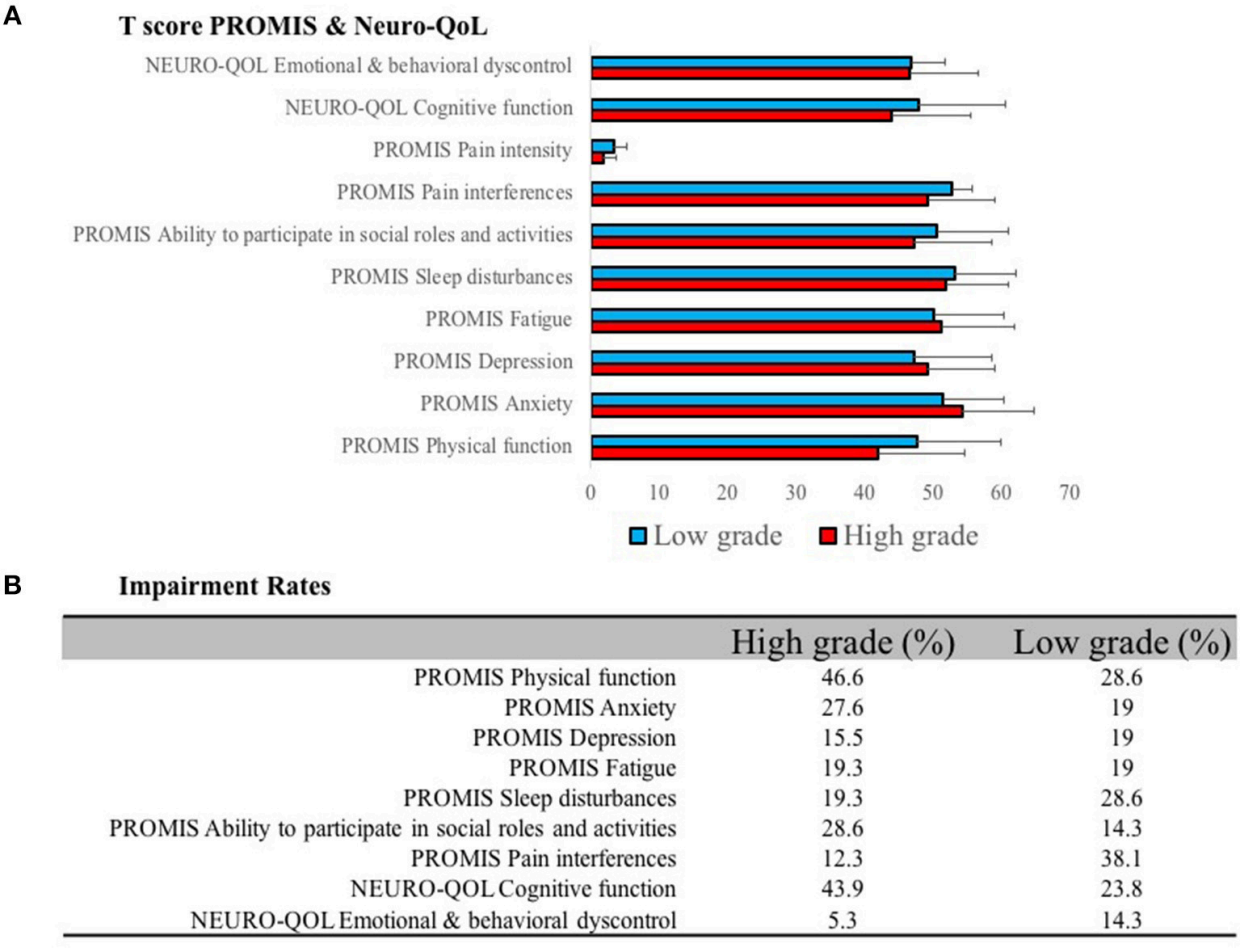
**(A)** PROMIS and Neuro-QoL domains were measured for low- and high-grade glioma patients. **(B)** Impairment rates were determined based on patients who scored >1 standard deviation beyond the normative mean.

Prevalence of impairment for the HGG cohort was elevated for PROMIS physical functioning (46.6%), NEURO-QOL cognitive dysfunction (43.9%), PROMIS ability to participate in social roles and activities (28.6%), and PROMIS anxiety (27.6%) (Figure 1). Clinical impairment rates for the LGG cohort were elevated for PROMIS pain interferences (38.1%), PROMIS physical functioning (28.6%), PROMIS sleep disturbance (28.6%), and NEURO-QOL cognition (23.8%). There were no significant differences between PROMIS and Neuro-QOL PRO scores between HGG and LGG groups, with the following exceptions: PROMIS pain intensity, in which patients with LGG experienced greater pain-related intensity relative to patients with HGG (*t*-score: HGG 1.76 ± 2, LGG 3.29 ± 3; *P* = 0.01) and greater distress from declining physical function among patients with HGG (*t*-score: HGG 41.83 ± 12.59, LGG 47.74 ± 12.16; *P* = 0.05) (Table 3).

**Table 3:** Comparison of HRQOL scores for low- and high-grade glioma groups.

**Variable**	**High-grade glioma (*****n*** **=** **58)**		**Low-grade glioma (*****n*** **=** **21)**		**η^2^**	***F*-value**	***P*-value**
	**Mean**	**SD**	**Mean**	**SD**			
Physical function	41.8	12.6	47.7	12.2	0.05	3.75	**0.05**
Anxiety	54.3	10.4	51.5	8.8	0.01	1.21	0.28
Depression	49.1	10.0	47.2	11.4	0.01	0.55	0.46
Fatigue	51.3	10.6	50.2	10.2	0.00	0.17	0.68
Sleep disturbances	52.01	9.2	53.2	9.0	0.00	0.23	0.64
Ability to participate in social roles and activities	47.2	11.4	50.7	10.4	0.02	1.44	0.23
Pain interferences	49.3	9.8	52.8	10.4	0.02	1.83	0.18
Pain intensity	1.8	2.0	3.3	3.0	0.08	6.72	**0.01**
Cognitive function	44.0	11.6	47.9	9.3	0.02	1.86	0.18
Emotional and behavioral dyscontrol	46.7	9.9	46.9	12.8	0.00	0.01	0.94

*SD, standard deviation. Bold values mean significant p value (<0.05)*.

### Aphasia Severity Impacts HRQOL Functional Domain

In a subgroup of 26 patients with dominant hemisphere gliomas within the perisylvian frontal, parietal, and temporal lobes, 7 had a BDAE aphasia severity score of 1–2 (severe) and 19 had a BDAE score of 3–5 (mild). The mean BDAE severity scores for both HGG and LGG cohorts were 3 ± 1 and 5 ± 0.5, respectively (*P* = 0.004). Aphasia severity had a moderate association with greater distress on PROMIS measures of anxiety (*r* = −0.51; *P* = 0.0074) and NEURO-QOL cognition (*r* = 0.55; *P* = 0.0033) (Table 4).

**Table 4:** Pearson correlation coefficients comparing the impact of aphasia on health-related quality of life functional and impairment domains.

	**Aphasia**
Physical function	0.09
Anxiety	**−0.51**
Depression	−0.31
Fatigue	−0.20
Sleep disturbances	−0.09
Ability to participate in social roles and activities	0.00
Pain interferences	−0.03
Pain intensity	−0.08
Cognition	**0.55**
Emotional and behavioral dyscontrol	−0.27

*Bold values mean significant p value (<0.05)*.

## Discussion

HRQOL measurements have become an increasingly important measure in the care of glioma patients. There is a need for reliable patient quality of life assessment measures which are easy to use and clinically relevant for both patients and clinicians. Assessing HRQOL PRO measures in glioma patients can be a challenge because of self-reporting difficulties in this population due to functional and cognitive impairments (25). The PROMIS survey was developed to measure PRO measures for patients with a variety of chronic diseases. PROMIS as a subjective assessment tool for glioma patients has been validated and compared to the more commonly used European Organization for Research and Treatment of Cancer (EORTC-30) and Caregiver Quality of Life Cancer (CQOLC) scales (26). To our knowledge, only one pilot study in a small cohort of 10 patients has described the use of PROMIS as a HRQOL assessment tool in adult high-grade glioma patients (26). Here we are the first to compare HRQOL using PROMIS and Neuro-QOL between adult LGG and HGG patients.

Perhaps unsurprisingly, 79.3% of all patients reported that they value quality of life over survival at the point of diagnosis. After cross sectional analysis of PROMIS and NEURO-QOL data, we found that LGG patients experienced more pain intensity and greater distress from declining physical function when compared with HGG patients. The biologic and psychological correlates to explain these differences are unclear; however, this information carries significance when caring for patients and determining clinical trial efficacy. Cognitive dysfunction is more commonly found in HGG patients; therefore distress measures in PRO domain such as pain intensity might be reported at different rates in LGG and HGG patients. We also found a high rate of impairment in the PROMIS functional domains assessed in our study population, again indicating that patient functional wellness should be carefully considered in an individualized treatment approach. Future studies may compare these prevalences to other cancer patient populations. Our results also demonstrate that aphasia severity is associated with increased anxiety and cognitive distress. We find a higher prevalence of severe aphasia in HGG patients relative to LGG, which may be due to selection bias due to small sample size or differences in intrinsic tumor biology.

Glioma patients suffer from a wide range of possible neurological and functional limitations which influences quality of life and survival. Aphasia and cognitive disorders are more prevalent in patients with WHO III and IV tumors. Cognitive dysfunction, as determined by global cognitive task performance, occurs in 35.9% of HGG patients and 23.7% of patients experience aphasia throughout their disease trajectory (27). It is therefore of little surprise that our Neuro-QoL analysis determined that 43.9% of HGG patients experience distress from impairment of cognitive function (Figure 1). Similar results are seen for distress from physical function in HGG patients. Despite the absence of identifiable oncological differences between our LGG and HGG cohorts, pain intensity scores were higher in LGG patients (Figure 1). This could be caused at least in part by the increased rate of cognitive dysfunction resulting in under reporting of pain in HGG patients. These differences bring to light important considerations when interpreting PRO in the adult glioma population. Looking beyond survival, when designing clinical trials, is critical given the extensive burden of symptoms experienced by glioma patients. Furthermore, it cannot be assumed that LGG and HGG patients experience the same symptoms and distress profile.

Patient reported outcome (PRO) measures are used in clinical practice as a mechanism to understand the natural history of disease or as a health measure of clinical change. There are few publications focused on thresholds constituting meaningful clinical change. Clinical judgment must be applied for the interpretation of clinically meaningful PRO. Defining the magnitude of change that is clinically important is necessary and there's a growing body of evidence for this important area of study. There are several terms for clinically relevant HRQOL change, including, minimally important difference (MID). “True” differences do not exist in HRQOL assessments and the magnitude of a score is an estimate which must be interpreted with clinical judgment (28). There is no empirical literature on which to base MID estimate; therefore, many use a half standard deviation (5 points on a T score metric). However clinical significance has been illustrated at a lower threshold (28, 29). MID for the adult glioma population are currently unavailable and a topic of future study. For example, patients with advanced stage cancer illustrate fatigue PROMIS MID of 3.0–5.0, pain interference MID of 4.0–6.0, and physical function MID of 4.0–6.0 (29). It is important to note that MID estimates vary based on cross sectional and longitudinal analysis. Furthermore, these assessments of clinical significance are averages across subjects; therefore, individual patients may require more or less to be clinically meaningful. The objective of this study was to evaluate adult glioma patient perspectives on functional domain affecting health related quality of life and apply cross sectional analysis of the PROMIS and Neuro-QOL instruments as subjective PRO measures in an adult low and high-grade glioma patient population. MID estimates were beyond the scope of this initial study which was focused on characterization of disease. Moving forward we hope to define MID and clinical relevance in the adult glioma population.

Other study limitations include the single institution small sample size which prohibited stratification of patients by additional potential confounders including tumor location, volume, or burden of disease at the time of assessment. Given our small sample size, within the LGG cohort we do not see the expected distribution across male and female patients. This difference does not reach statistical difference; however, it's not in line with expected results for the general population (30). Gender differences may contribute to variations in health outcomes. These differences in PRO have been reported primarily with pain and pain related disorders; however, it is certainly possible that gender differences impact this dataset focused on adult glioma patients (31). Furthermore, pain intensity interpretation is limited given that PROMIS is specifically focused on global pain making the distinction between headaches and neuropathy impossible. It is well known that both patient perspectives and HRQOL PRO measures vary with time (32). For this reason, our current analysis focused solely on HRQOL at the time of initial diagnosis with the goal of longitudinal analysis throughout the course of disease to better understand how responses change with time. This and other limitations will be mitigated by increasing the sample size in future studies. Additionally, while subjective patient PRO measures are valuable, it should be noted that they are excellent HRQOL measures of distress but not dysfunction (25). Objective measures of function should also be incorporated into patient assessment.

## Conclusions

HRQOL measurements have become increasingly important in glioma research and clinical practice. There has been limited and slow progress in developing effective treatments for glioma patients. The natural history of the disease in addition to treatment related side-effects can also negatively impact patient function and HRQOL. Treatment of glioma patients should focus on both prolonging life in addition to maintaining quality of life. The PROMIS and NEURO-QOL are two measures, which are valuable for quantifying patient reported HRQOL. The current study will hopefully lead to the use of these tools in more robust clinical research and practice settings.

## Ethics Statement

This study was performed in accordance with the University of Michigan institutional ethics committee (IRB- HUM00092238). The protocol was approved by the UM-IRB and all subjects gave written informed consent in accordance with the Declaration of Helsinki.

## Author Contributions

NG: manuscript preparation, data analysis, data interpretation; DA, NB, EB, LW: manuscript preparation, data analysis; AB, ZM: data acquisition, data analysis; KK: data acquisition, manuscript preparation; TF, KM: data acquisition; SS, NC: concept, study design, manuscript preparation; SH-J: concept, study design, data acquisition, data analysis, manuscript preparation, manuscript editing.

### Conflict of Interest Statement

The authors declare that the research was conducted in the absence of any commercial or financial relationships that could be construed as a potential conflict of interest.

## References

[1] Hervey-Jumper SL Li J Lau D Molinaro AM Perry DW Meng L. Awake craniotomy to maximize glioma resection: methods and technical nuances over a 27-year period. J Neurosurg. (2015) 123:325–39. 10.3171/2014.10.JNS141520 25909573

[2] Rouse C Gittleman H Ostrom QT Kruchko C Barnholtz-Sloan JS. Years of potential life lost for brain and CNS tumors relative to other cancers in adults in the United States, 2010. Neuro Oncol. (2016) 18:70–7. 10.1093/neuonc/nov249 26459813 PMC4677421

[3] Perez L Huang J Jansky L Nowinski C Victorson D Peterman A. Using focus groups to inform the neuro-QOL measurement tool: exploring patient-centered, health-related quality of life concepts across neurological conditions. J Neurosci Nurs. (2007) 39:342–53. 10.1097/01376517-200712000-00005 18186419

[4] Cella DF. Methods and problems in measuring quality of life. Support Care Cancer. (1995) 3:11–22. 10.1007/BF00343916 7697298

[5] Cella DF Bonomi AE. Measuring quality of life: 1995 update. Oncology (Williston Park). (1995) 9:47–60. 8608056

[6] Halkett GKB Lobb EA Rogers MM Shaw T Long AP Wheeler HR. Predictors of distress and poorer quality of life in high grade Glioma patients. Patient Educ Couns. (2015) 98:525–32. 10.1016/j.pec.2015.01.002 25638306

[7] Giovagnoli AR. Quality of life in patients with stable disease after surgery, radiotherapy, and chemotherapy for malignant brain tumour. J Neurol Neurosurg Psychiatry. (1999) 67:358–63. 10.1136/jnnp.67.3.358 10449559 PMC1736531

[8] Scheibel RS Meyers CA Levin VA. Cognitive dysfunction following surgery for intracerebral glioma: influence of histopathology, lesion location, and treatment. J Neurooncol. (1996) 30:61–9. 10.1007/BF00177444 8865004

[9] Weitzner MA Meyers CA. Cognitive functioning and quality of life in malignant glioma patients: a review of the literature. Psychooncology. (1997) 6:169–77. 10.1002/(SICI)1099-1611(199709)6:3<169::AID-PON269>3.0.CO;2-# 9313282

[10] Nasreddine ZS Phillips NA Bédirian V Charbonneau S Whitehead V Collin I. The Montreal Cognitive Assessment, MoCA: a brief screening tool for mild cognitive impairment. J Am Geriatr Soc. (2005) 53:695–9. 10.1111/j.1532-5415.2005.53221.x 15817019

[11] Olson RA Chhanabhai T McKenzie M. Feasibility study of the Montreal Cognitive Assessment (MoCA) in patients with brain metastases. Support Care Cancer. (2008) 16:1273–8. 10.1007/s00520-008-0431-3 18335256

[12] Olson RA Iverson GL Carolan H Parkinson M Brooks BL McKenzie M. Prospective comparison of two cognitive screening tests: diagnostic accuracy and correlation with community integration and quality of life. J Neurooncol. (2011) 105:337–44. 10.1007/s11060-011-0595-4 21520004

[13] Olson R Tyldesley S Carolan H Parkinson M Chhanabhai T McKenzie M. Prospective comparison of the prognostic utility of the mini mental state examination and the montreal cognitive assessment in patients with brain metastases. Support Care Cancer. (2011) 19:1849–55. 10.1007/s00520-010-1028-1 20957394

[14] Mortensen GL Jakobsen JK. Patient perspectives on quality of life after penile cancer. Dan Med J. (2013) 60:A4655. 23809966

[15] Singer PA Martin DK Kelner M. Quality end-of-life care: patients' perspectives. JAMA. (1999) 281:163–8. 10.1001/jama.281.2.163 9917120

[16] Cook KF Jensen SE Schalet BD Beaumont JL Amtmann D Czajkowski S. PROMIS measures of pain, fatigue, negative affect, physical function, and social function demonstrated clinical validity across a range of chronic conditions. J Clin Epidemiol. (2016) 73:89–102. 10.1016/j.jclinepi.2015.08.038 26952842 PMC5131708

[17] Bode RK Hahn EA DeVellis R Cella D Patient-Reported Outcomes Measurement Information System social domain working group. Measuring participation: the patient-reported outcomes measurement information system experience. Arch Phys Med Rehabil. (2010) 91:S60–5. 10.1016/j.apmr.2009.10.035 20801282 PMC3671872

[18] Cella D Yount S Rothrock N Gershon R Cook K Reeve B. The Patient-Reported Outcomes Measurement Information System (PROMIS): progress of an NIH Roadmap cooperative group during its first two years. Med Care. (2007) 45:S3–11. 10.1097/01.mlr.0000258615.42478.55 17443116 PMC2829758

[19] Quach CW Langer MM Chen RC Thissen D Usinger DS Emerson MA. Reliability and validity of PROMIS measures administered by telephone interview in a longitudinal localized prostate cancer study. Qual Life Res. (2016) 25:2811–23. 10.1007/s11136-016-1325-3 27240448 PMC6126915

[20] Cella D Riley W Stone A Rothrock N Reeve B Yount S. The Patient-Reported Outcomes Measurement Information System (PROMIS) developed and tested its first wave of adult self-reported health outcome item banks: 2005-2008. J Clin Epidemiol. (2010) 63:1179–94. 10.1016/j.jclinepi.2010.04.011 20685078 PMC2965562

[21] Salsman JM Victorson D Choi SW Peterman AH Heinemann AW Nowinski C. Development and validation of the positive affect and well-being scale for the neurology quality of life (Neuro-QOL) measurement system. Qual Life Res. (2013) 22:2569–80. 10.1007/s11136-013-0382-0 23526093 PMC3855608

[22] Chang EF Raygor KP Berger MS. Contemporary model of language organization: an overview for neurosurgeons. J Neurosurg. (2015) 122:250–61. 10.3171/2014.10.JNS132647 25423277

[23] Wilson SM Lam D Babiak MC Perry DW Shih T Hess CP. Transient aphasias after left hemisphere resective surgery. J Neurosurg. (2015) 123:581–93. 10.3171/2015.4.JNS141962 26115463 PMC4558229

[24] Cohen J Statistical Power Analysis for the Behavioral Sciences. 2nd ed. Hillsdale, NJ: L. Erlbaum Associates (1988).

[25] Hickmann AK Hechtner M Nadji-Ohl M Janko M Reuter AK Kohlmann K. Evaluating patients for psychosocial distress and supportive care needs based on health-related quality of life in primary brain tumors: a prospective multicenter analysis of patients with gliomas in an outpatient setting. J Neurooncol. (2017) 131:135–51. 10.1007/s11060-016-2280-0 27638638

[26] Romero MM Flood LS Gasiewicz NK Rovin R Conklin S. Validation of the national institutes of health patient-reported outcomes measurement information system survey as a quality-of-life instrument for patients with malignant brain tumors and their caregivers. Nurs Clin North Am. (2015) 50:679–90. 10.1016/j.cnur.2015.07.009 26596656

[27] IJzerman-Korevaar M Snijders TJ de Graeff A Teunissen SCCM de Vos FYF. Prevalence of symptoms in glioma patients throughout the disease trajectory: a systematic review. J Neurooncol. (2018) 140:485–96. 10.1007/s11060-018-03015-9 30377935 PMC6267240

[28] Kozlowski AJ Cella D Nitsch KP Heinemann AW. Evaluating individual change with the quality of life in neurological disorders (neuro-QoL) short forms. Arch Phys Med Rehabil. (2016) 97:650–4.e8. 10.1016/j.apmr.2015.12.010 26740062 PMC4994512

[29] Yost KJ Eton DT Garcia SF Cella D. Minimally important differences were estimated for six patient-reported outcomes measurement information system-cancer scales in advanced-stage cancer patients. J Clin Epidemiol. (2011) 64:507–16. 10.1016/j.jclinepi.2010.11.018 21447427 PMC3076200

[30] Ostrom QT Gittleman H Truitt G Boscia A Kruchko C Barnholtz-Sloan JS. CBTRUS statistical report: primary brain and other central nervous system tumors diagnosed in the United States in 2011-2015. Neuro Oncol. (2018) 20:iv1–86. 10.1093/neuonc/noy131 30445539 PMC6129949

[31] Chow S Ding K Wan BA Brundage M Meyer RM Nabid A. Gender differences in pain and patient reported outcomes: a secondary analysis of the NCIC CTG SC. 23 randomized trial. Ann Palliat Med. (2017) 6:S185–94. 10.21037/apm.2017.08.12 29156903

[32] Sagberg LM Solheim O Jakola AS. Quality of survival the 1st year with glioblastoma: a longitudinal study of patient-reported quality of life. J Neurosurg. (2016) 124:989–97. 10.3171/2015.4.JNS15194 26430849

